# Recent advances in understanding endocrine disruptors: DDT and related compounds

**DOI:** 10.12703/b/9-7

**Published:** 2020-11-12

**Authors:** Stephen Safe

**Affiliations:** 1 Department of Veterinary Physiology & Pharmacology, Texas A&M University, Texas, USA

**Keywords:** Endocrine disrupting-compound, DDT, DDE

## Abstract

Endocrine-disrupting compounds (EDCs) are environmental contaminants that modulate estrogen, androgen, and thyroid hormone receptor signaling and it has been hypothesized that human exposures to EDCs induce multiple adverse health effects. Some of these responses include male and female reproductive tract problems, obesity, and neurological/neurobehavior deficits. Extensive laboratory animal and some human studies support the EDC hypothesis. However, there is a debate among scientists and regulators regarding the adverse human health impacts of EDCs and this review highlights and gives examples of some of the concerns.

## Introduction

The concern regarding endocrine-disrupting compounds (EDCs) and their potential adverse health impacts originated from a few studies that highlighted or hypothesized a limited number of health problems that could be caused by this class of compounds^1^. Pharmacological doses of diethylstilbestrol (DES), a potent estrogenic drug administered to pregnant women, induced a multitude of reproductive tract problems in female and male offspring, and results of laboratory animal studies observed many of the same toxic effects. The studies on DES and its effects were instrumental in raising concerns regarding possible endocrine-mediated toxicities. Meta-analysis of global sperm counts from many locations over the 1938–1991 period suggested that there was a time-dependent decrease in sperm counts from 1938 (113 × 10^6^/mL) to 1991 (66 × 10^6^/mL), and it was hypothesized that decreased sperm counts and other male reproductive tract disorders were linked to exposures to estrogenic compounds. It was also hypothesized that environmental estrogens, particularly some organochlorine pesticides, may contribute to increases in the incidence of breast cancer in women^2^. These observations, coupled with some studies showing increases in other hormone-dependent abnormalities (that is, cryptorchidism and hypospadias), resulted in the initial focus on estrogenic EDCs and this was later expanded to EDCs that disrupt androgen and thyroid hormone receptor signaling pathways. Subsequent studies examining breast cancer and sperm counts have questioned the validity of some of the early studies addressing the estrogen hypothesis (reviewed in 3). There is also evidence showing the complexity of the problem and the difficulties in affirming or negating the role of EDCs in affecting breast cancer, sperm counts and other male reproductive tract problems, and many other health issues, and some of these issues will be discussed below.

## Ongoing studies

In the last two decades, laboratory animal and human studies on EDCs have significantly expanded in terms of not only the number of publications but also the hypothesized number of health problems associated with exposure to EDCs. A search of PubMed for articles on “endocrine disruptors” lists over 9500 publications and all but 84 of these articles have been published since 2000. From the original focus on sperm counts, breast cancer, hypospadias, and cryptorchidism, various scientific groups, professional societies, and regulatory agencies have expanded the health effects of concern to include multiple diseases. For example, a statement issued by the Endocrine Society^4^ indicates that EDCs may contribute to multiple diseases, which include the following: obesity, diabetes mellitus type 2, and cardiovascular disease; female reproductive tract problems (“abnormal puberty, irregular cyclicity, reduced fertility, infertility, polycystic ovarian syndrome, endometriosis, fibroids, preterm births and adverse birth outcomes”); male reproductive tract problems (genitalia malformations, cryptorchidism and hypospadias, testicular cancer, and semen quality); hormone-sensitive cancers (breast, ovary, and uterus); thyroid disruption; and neurodevelopmental and neuroendocrine effects (IQ and adverse neurodevelopmental, neurocognitive, and neurobehavioral outcomes). This list of health problems is more extensive than those proposed by the World Health Organization and was based, in part, on laboratory animal and human epidemiological studies, some of which are controversial and less than convincing. Nevertheless, the Executive Summary of the EDC2 report published by the Endocrine Society^4^ states that the “data reviewed in EDC2 removes any doubt that EDCs are contributing to increased chronic disease burdens related to obesity, diabetes mellitus, reproduction, thyroid cancers and neuroendocrine and neurodevelopmental functions”. It should also be pointed out that a major concern regarding the potential adverse health impact of EDCs focuses on the timing of exposure where significant adverse health effects of EDCs may be linked to *in utero* and early postnatal exposures: The Developmental Origins of Health and Disease (DOHaD) hypothesis emphasizes that many adverse health effects observed later in life are due, in part, to exposures (diet and chemicals) during critical periods of early development^5^. Thus, a major emphasis on EDC research has focused on correlating early-life exposures to EDCs with adverse health outcomes that develop later in life. These are difficult studies since validated maternal exposure data are sparse and usually involve only one or two samplings (for example, urine and blood) of pregnant women to estimate *in utero* exposures which then are correlated with various adverse effects in offspring. The DOHaD hypothesis is supported by some laboratory animal and human studies and is an important area of research which will benefit from future results of ongoing prospective studies.

Despite the proposed long list of diseases that may be impacted by exposure to one or more EDCs, many of these claims have been supported or disputed by individual scientists and scientific societies^6,7^. Fitzgerald^8^ recently evaluated shortcomings with respect to human studies and the subsequent problems associated with correlating EDC exposure data with human diseases. That article is not judgmental in terms of whether exposure to EDCs induces adverse health effects but emphasizes the need for international cooperation and collaboration to address the issue. It is also pointed out that “generating reliable long-term data for human diseases putatively associated with EDCs has not yet been achieved”^8^.

**Figure 1.**
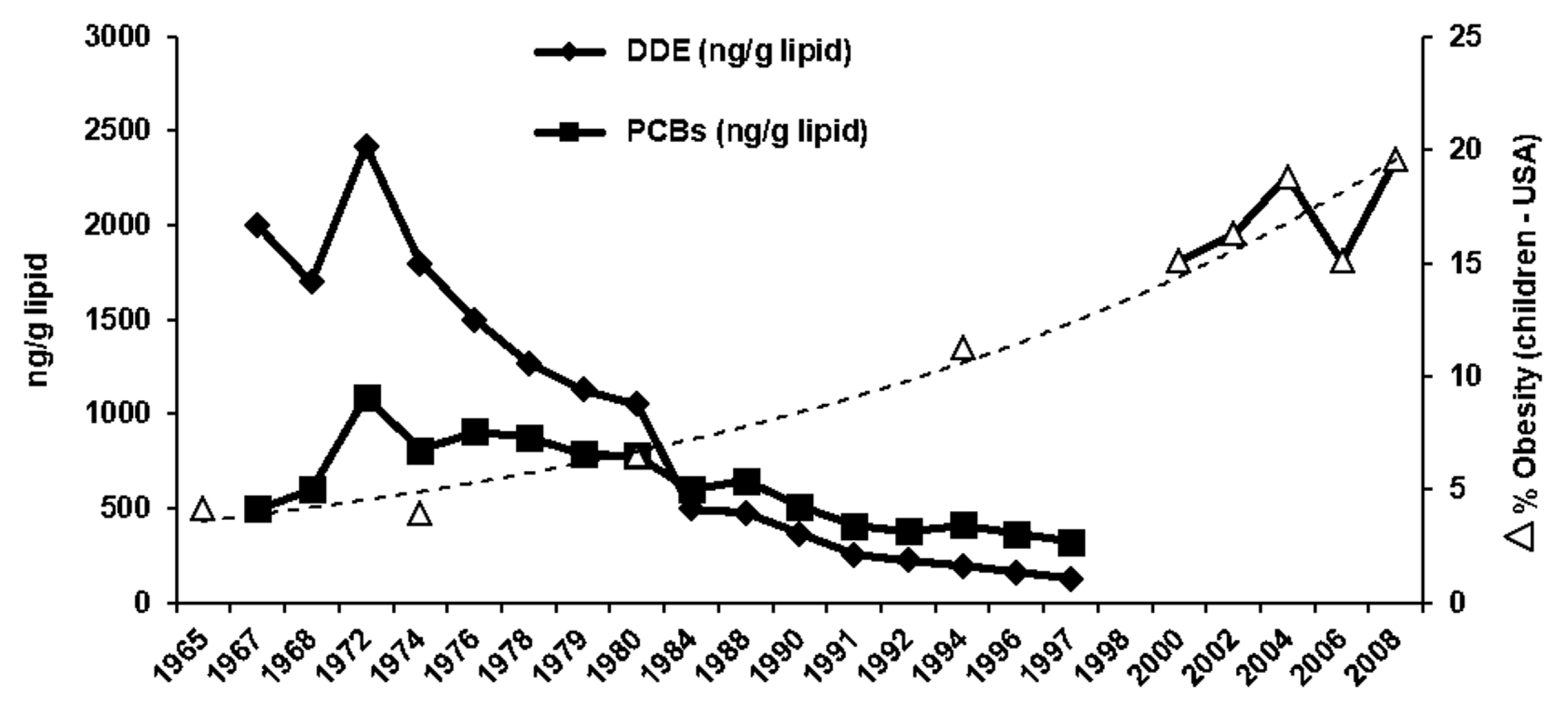
Time-dependent changes in dichlorodiphenyl dichloroethene (DDE) (♦) and total polychlorinated biphenyl (PCB) (■) levels in human milk^21^ and time-dependent increases in percentage of obesity (Δ) of 6- to 11-year-olds in the US^21^.

One example of an EDC response that has been extensively investigated is the effect of the pesticide p,p′-DDT and its persistent metabolite p,p′-DDE on obesity, which is an important risk factor for other hypothesized EDC-induced responses. Despite the banning of DDT use in most but not all countries (for example, India), the highly persistent p,p′-DDE metabolite is still detectable in most serum and adipose tissue samples^9^. There was an initial concern regarding the possible role of DDT (that is, p,p′-DDE) as a factor that contributes to the development of breast cancer; however, this is not supported by global studies correlating DDE levels and breast cancer^10^. Most of the reports correlating p,p′-DDE levels and breast cancer were case-control studies; however, a recent prospective nested case-control study indicated that higher p,p′-DDT exposure before puberty was associated with increased rates of breast cancer later in life and this approach is clearly of interest^11^. A recent review article analyzed the association between exposure to p,p′-DDT and p,p′-DDE with obesity, and the authors identified and analyzed seven prospective studies showing an association between levels of exposure to p,p′-DDE and body mass index^12^. These results, coupled with laboratory animal studies, led the authors to conclude “that DDT and DDE exposures during the developmental period can be classified as presumed human obesogens”^12^. Several recent studies both support and question the role of DDE and obesity. Maternal exposure to o,p′-DDT was associated with several parameters of obesity in a prospective birth cohort 53 years after initial sampling (1959–1967) but these associations were not observed for p,p′-DDE^13^. Early-life exposure to DDE and other persistent organic pollutants (POPs) did not show any association with body mass index in 5-year-old Faroese children^14^, whereas in a cohort of newborns from the Canary Islands, DDE but not other POPs was associated with increased birth weights but only in girls^15^. Other studies showed that current exposures to DDE were associated with obesity-related health problems^16–18^, and it was also reported that DDE induced changes in the microbiota (rat) and that this may also need to be considered^19^. Some additional puzzling aspects of the obesity studies and the role of p,p′-DDE as an impactful EDC include the following:

1. In most Western countries, p,p′-DDE levels have decreased by up to 90% over the past 30 to 40 years^9^.

2. While the p,p′-DDE levels have been decreasing in the US and other Western countries, there has been a dramatic increase in obesity. Thus, there is an inverse association between decreasing DDE levels and increasing obesity in the US and some other developed countries^20^.

3. Figure 1 illustrates an example of the decrease in polychlorinated biphenyls (PCB) and DDE levels in human milk sample in Sweden from 1965 to 1997^21^ and this pattern of time-dependent decreases in these organochlorine compounds is observed in the US and most developed countries. In contrast, while organochlorine contaminant levels are decreasing, there was an inverse time-dependent increase in the percentage of obesity of 6- to 11-year-olds in the US^22^.

4. Many other countries, such as Japan, Korea, and China, where average DDE levels in humans^9^ are similar (204, 232, and 193 ng/g lipid, respectively) to those observed in the US (165 ng/g lipid)^9^, are not considered to be “obese” countries. The overall percentages of the population that is obese in the US, Japan, Korea, and China are 33.7%, 3.3%, 5.8%, and 6.9%, respectively, with world obesity rankings of 19, 185, 166, and 157, respectively, among 190 countries surveyed. Thus, although DDE levels are similar in all four countries, their obesity levels as did not correlate with DDE levels and are probably related to diet. Moreover, a comparison of DDE levels and obesity in the US and India is more dramatic. India has high levels of DDE (2654 ng/g lipid) because of continued use of the pesticide but ranks 174 (4.9%) out of 190 countries in terms of percentage of the population that is obese. In contrast, levels of DDE are significantly lower in the US (165 ng/g lipid) compared with those observed in India but the percentage of obese individuals is much higher (33.7%). These correlations also apply for mean body mass index in these same countries.

This commentary illustrates that although it has been hypothesized that EDCs such as DDT and related compounds contribute to many health problems (including obesity and obesity-related diseases), many issues and problems need to be considered in order to scientifically assess the extent of EDC-induced health problems. These problems include the quality of the human data and the integration of global differences in rates of disease and levels of exposure to EDCs. Moreover, the evaluation of the combined effects of multiple EDCs is an important consideration that needs to be evaluated. A recent report has summarized the development of an approach for evaluating and assessing hazard identification of EDCs, and its recommendations will need to be evaluated^23^.
